# Combinational Pulsing of TAAs Enforces Dendritic Cell-Based Immunotherapy through T-Cell Proliferation and Interferon-γ Secretion in LLC1 Mouse Model

**DOI:** 10.3390/cancers16020409

**Published:** 2024-01-18

**Authors:** Jae-Ung Lee, Sang-Heon Kim, Sung-Hoon Lee, Min-Jae Ji, Jeong-Ah Jin, Hyung-Joon So, Myoung-Lim Song, Hong-Ki Lee, Tae-Wook Kang

**Affiliations:** 1 Institute of Cell Biology and Regenerative Medicine, EHLBio Co., Ltd., Uiwang-si 16006, Republic of Korea; lju4815@gmail.com (J.-U.L.); ksh_8959@naver.com (S.-H.K.);; 2 EHLCell Clinic, Seoul 06029, Republic of Korea

**Keywords:** dendritic cells, tumor-associated antigen, non-small cell lung cancer, immunotherapy

## Abstract

**Simple Summary:**

Despite the proven efficacy and confirmed safety of dendritic cell therapy, the limited response against cancer remains a challenge. This study aimed to investigate the potential of combinational tumor-associated antigens (TAAs) pulsing to enhance the antigen-presenting ability of dendritic cells. TAAs-pulsed DCs demonstrated anti-cancer capabilities, including tumor growth suppression, increased proliferation of splenic T cells, and elevated IFN-γ secretion. While the combinational pulsing approach shows promise in addressing the limitations of dendritic cell therapies, additional research is required to explore diverse combinations of TAAs for potential therapeutic applications.

**Abstract:**

NSCLC, the most common type of lung cancer, is often diagnosed late due to minimal early symptoms. Its high risk of recurrence or metastasis post-chemotherapy makes DC-based immunotherapy a promising strategy, offering targeted cancer destruction, low side effects, memory formation, and overcoming the immune evasive ability of cancers. However, the limited response to DCs pulsed with single antigens remains a significant challenge. To overcome this, we enhanced DC antigen presentation by pulsing with TAAs. Our study focused on enhancing DC-mediated immune response specificity and intensity by combinatorial pulsing of TAAs, selected for their prevalence in NSCLC. We selected four types of TAAs expressed in NSCLC and pulsed DCs with the optimal combination. Next, we administered TAAs-pulsed DCs into the LLC1 mouse model to evaluate their anti-tumor efficacy. Our results showed that TAAs-pulsed DCs significantly reduced tumor size and promoted apoptosis in tumor tissue. Moreover, TAAs-pulsed DCs significantly increased total T cells in the spleen compared to the unpulsed DCs. Additionally, in vitro stimulation of splenocytes from the TAAs-pulsed DCs showed notable T-cell proliferation and increased IFN-γ secretion. Our findings demonstrate the potential of multiple TAA pulsing to enhance the antigen-presenting capacity of DCs, thereby strengthening the immune response against tumors.

## 1. Introduction

Lung cancer is known to be one of the most commonly diagnosed cancers in people aged 65 or older and the second most commonly diagnosed in both men and women. Lung cancer can be divided into small cell lung cancer (SCLC) and non-small cell lung cancer (NSCLC), with approximately 85% of lung cancer patients classified as non-small cell lung cancer. Early-diagnosed stage I lung cancer has a 5-year survival rate of 68.4%, while the survival rate for stage IV lung cancer is known to be very low at 5.8%, with the majority of patients being diagnosed at stage IV and overall survival rates being very low [1]. Despite this, the low early diagnosis rate of lung cancer serves as a major factor in decreasing the overall survival rate of lung cancer patients.

There are several treatment approaches for lung cancer, including surgery, radiotherapy, chemotherapy, and targeted therapy. Radiation therapy is a treatment that uses high doses of radiation to kill cancer cells and shrink tumor tissues and is frequently administered after surgical resection. However, it was reported that the efficacy of radiation therapy in NSCLC can be potentially diminished due to tumor microenvironmental factors, such as cellular radiotherapy resistance, hypoxia-related radiation sensitivity decreases, and tumor localization complexities [2].

Chemotherapy is the most common cancer treatment that uses drugs to induce DNA damage or inhibit cell division, leading to cancer cell death. However, a significant portion of NSCLC patients is gradually developing resistance to drugs as chemotherapy accumulates. This resistance poses challenges in maintaining the therapeutic effects of drugs over an extended period. Despite adjunctive chemotherapy following surgical resection, it was reported that approximately 50% of NSCLC stage IB patients and 75% of stage IIIA patients experience recurrence and metastasis [3]. Both radiation therapy and chemotherapy not only target the cancerous tissues but also damage a patient’s normal tissues, leading to various side effects, such as fatigue, nausea, and hair loss. To minimize these side effects and enhance anti-cancer efficacy, targeted therapy has been increasingly explored in recent times.

Targeted therapy is a specialized cancer treatment that uses drugs to target specific mutant proteins, such as EGFR, ALK, and ROS1, which drive the growth of cancer cells [4]. However, NSCLC patients who underwent targeted therapy exhibited a high response rate and improved overall survival rate. However, a limitation arises from the fact that the subset of patients with targetable specific mutant proteins constitutes a small proportion of the overall population (approximately 20%) [5,6].

Anti-cancer immunotherapies can be classified based on their mechanisms of action and characteristics as follows: (1) therapies that utilize the immune cells to directly target and kill cancer cells (e.g., NK cell therapy) [7]; (2) methods that inhibit the immune evasion mechanisms of cancer cells (e.g., immune checkpoint inhibitors, such as anti-CTLA-4 and anti-PD-1 antibodies) [8]; (3) approaches that employ genetically engineered immune cells tailored to recognize cancer-specific antigens (e.g., CAR-T therapy) [9]; (4) therapy that uses cancer antigens to enhance the patient’s own immune response against specific antigens (e.g., dendritic cell-based therapy) [10].

Dendritic cells serve as pivotal orchestrators bridging the innate and adaptive immune responses. Cancer cells generate mutated proteins, and a subset of these proteins is acquired by dendritic cells as cancer-specific antigens. Then, dendritic cells can activate CD4^+^ T cells through the presentation of MHC II molecules while the cross-presentation ability of dendric cells directly activates CD8^+^ T cells by presenting MHC I molecules. These mechanisms can enhance the activation of immune responses and amplify the attack against cancer cells [11]. However, the immunosuppressive microenvironment of tumors can weaken the antigen-presentation mechanism of dendritic cells and their interactions with other immune cells [12]. For these reasons, therapeutic approaches are being attempted through in vitro cultivation to load tumor-associated antigens (TAAs) onto dendritic cells and aiming to effectively respond to cancer cells.

TAAs can be expressed differently depending on the type of cancer and a single tumor may express multiple types of TAAs simultaneously [13]. Moreover, even within the same cancer type, there can be slight variations among individual patients. Due to the expression features of tumor-associated antigens (TAAs), we hypothesized that loading multiple types of TAAs onto dendritic cells could enhance their anti-cancer therapeutic efficacy. Four different NSCLC-related TAAs (WT-1, MUC-1, Survivin/BIRC5, and TERT) were selected [14,15,16,17,18]. All these TAAs are strongly associated with tumor growth, and each plays specific roles contributing to cancer progression. For instance, WT-1, widely used as an antigen for anti-cancer immunotherapy, is expressed not only in various cancer types but also in cancer-surrounding endothelial cells, stimulating angiogenesis [19,20]. MUC-1 provides a sustained survival signal to tumors [21]; BIRC5 is known to be involved in drug resistance [22]; and TERT is reactivated in approximately 90% of primary tumors, including NSCLC [23,24].

In this study, we aimed to evaluate the multiple TAAs-pulsed dendritic cell-based immunotherapeutic efficacy in vivo. For this purpose, we selected four TAAs commonly expressed in NSCLC and formulated a TAA cocktail. We established a mouse model transplanted with LLC1, the mouse-derived NSCLS cell line, and examined the effects on tumor growth inhibition and activation of immune cells following the administration of TAAs-pulsed dendritic cells.

## 2. Materials and Methods

### 2.1. Materials

WT-1, BIRC5, MUC-1, and TERT were purchased from MyBioSource (San Diego, CA, USA). Recombinant mGM-CSF was purchased from PeproTech (Cranbury, NJ, USA). Cisplatin and lipopolysaccharide (LPS) were purchased from Sigma-Aldrich (Burlington, MA, USA). Fluorophore-labeled monoclonal antibodies (Annexin V-FITC, 7-AAD, CD3e-FITC, CD4-PE, CD8-Pacific blue, CD11c-APC, CD80-PE, CD86-FITC, Fixable Viability Stain 780, IFN-γ-BV605, MHC I-PE, MHC II-PerCP-Cy5.5) were purchased from BD Bioscience (Franklin Lakes, NJ, USA). Cell trace violet was purchased from Invitrogen (Waltham, MA, USA). LLC1 was purchased from ATCC (Manassas, VA, USA).

### 2.2. Isolation and Generation of TAAs-Pulsed Dendritic Cells

Bone marrow cells were isolated from the femur and tibia of 6- to 8-week-old C57BL/6 mice. Erythrocytes were lysed using ACK lysis buffer and filtered through a 100 μm cell strainer. Cells were collected and suspended with DC differentiation media (RPMI-1640 containing 10% FBS, 20 nM of penicillin/streptomycin, and 20 ng/mL of recombinant mouse granulocyte-macrophage colony-stimulating factor (rmGM-SCF)). Then, 3 × 10^6^ cells/well were incubated in a 6-well plate for 7 days at 37 °C, 5% CO_2_. On day 3 and day 5, fresh medium was added. On day 6, the fresh medium was half-changed. On day 7, differentiated immature dendritic cells (imDCs) were harvested. Differentiated imDCs were stimulated with 100 ng/mL of TLR4 ligand lipopolysaccharide (LPS) for another 16 h. Matured DCs (mDCs) were then pulsed with various concentrations of tumor-associated antigens (TAAs) for 3 h at 37 °C, 5% CO_2_.

### 2.3. Characterization

About 5 × 10^5^ cells of imDCs and mDCs were stained with specific surface markers (CD80, CD86, MHC I, MHC II) for 20 min at room temperature in the dark, and the cells were analyzed using a flow cytometer FACSVerse™ (BD, Franklin Lakes, NJ, USA), stained imDCs and mDCs were used as a negative control.

### 2.4. Apoptosis and Necrosis Assay

The cytotoxicity of various concentrations of the TAAs mixture was analyzed by Annexin V/7-AAD staining. TAAs-pulsed DCs were stained with Annexin V/7-AAD and analyzed by a flow cytometer (BD, FACS verse). The percentage of viable cells (Annexin V^−^/7-AAD^−^), early apoptotic cells (Annexin-V^+^/7-AAD^−^), and necrotic cells (7-AAD^+^) were analyzed in each experiment. H_2_O_2_ was treated as a positive control.

### 2.5. Splenic T-Cell Population Analysis

Five mice were randomly selected from each group and each spleen from the mice was extracted. Spleens were prepared as a single-cell suspension by homogenization, and erythrocytes were lysed with ACK lysis buffer (Thermo fisher scientific, Waltham, MA, USA). 1 × 10^6^ splenocytes were stimulated with PMA/Ionomycin and Golgistop reagent for 5 h. Cells were stained with T-cell-specific markers (CD3e, CD4, CD8). Stained cells were fixed with fixation solution for 20 min at 4 °C. Then, fixed cells were washed twice with perm/wash buffer and stained with an IFN-γ-specific intracellular marker for another 30 min at 4 °C in the dark. After the final washing step, cells were resuspended and analyzed using a flow cytometer (BD, FACS verse).

### 2.6. T-Cell Proliferation and IFN-γ ELISA Assay

To analyze T-cell proliferation, 1 × 10^6^ splenocytes were stimulated with PMA/Ionomycin for 72 h after being stained with Cell Trace Violet (CTV), and cells were analyzed using a flow cytometer (BD, FACS verse). Supernatants after stimulation were collected and stored at −70 °C for cytokine quantification. To measure the concentration of T-cell-secreted IFN-γ, we performed enzyme-linked immunosorbent assays (ELISA) according to the manufacturer’s instructions.

### 2.7. Animal Management

This study was conducted at a GLP-certified institute Biotoxtech Co., Ltd. (Cheonwon-gun, Chungcheongbuk-do, South Korea). Five-week-old male C57BL/6 mice were purchased from ORIENTBIO Inc. (Seongnam-si, Gyeonggi-do, South Korea). The examinations were initiated when the mice reached 7 weeks of age. Musculus lung carcinoma cell line (LLC1) cells were cultured in DMEM containing 10% FBS, penicillin–streptomycin. Cells were harvested and resuspended at a concentration of 3 × 10^6^ cells/mL in RPMI-1640 serum-free media. Then, cells were mixed with Matrigel at a 1:1 ratio and subcutaneously injected into the right flank of male C57BL/6 mice. After the LLC1 injection, body weight was monitored daily. And the tumor size was calculated three times a week using the formula TV (tumor volume, mm^3^) = L (long axis, mm) × S2 (short axis, mm^2^) × 1/2. After the tumor volume reached 80 to 150 mm^3^, mice were randomly divided into four groups. About 1 × 10^6^ DC cells resuspended in normal saline were subcutaneously injected into the inguinal region twice a week for 2 weeks. The same volume of normal saline was injected intravenously as a negative control and 2 mg/kg of Cisplatin was injected intravenously as a positive control. When the tumor volume of the negative control group reached 1500 mm^3^, mice were sacrificed and analyzed.

### 2.8. Histology

Extracted tumors were fixed in 10% neutral buffered formalin, prepared as paraffin blocks, and sectioned to a thickness of 5 μm. Slide glasses with attached tumor sections were stained for hematoxylin and eosin (H&E) or TUNEL assay. Stained tissues were observed using microscopy.

The necrosis score was evaluated based on the extent of necrosis across the entire cross-section of the tumor. The necrosis was determined by morphological changes such as nuclear collapse or loss and cytoplasmic eosinophilia. In the TUNEL assay, brown-stained nuclei were considered as positive cells, and the number of positive cells was expressed as a percentage of the total cell count.

### 2.9. Statistical Analysis

The statistical analysis was performed using GraphPad Prism 8.0 (U.S.A). Student *t*-test was utilized for comparing two different experimental groups. Data were presented as the mean ± SD, and values less than 0.05 were considered statistically significant.

## 3. Results

### 3.1. Cytotoxicity Test for Combinational TAAs Pulsing

Bone marrow progenitor cells were isolated from the femur and tibia of C57BL/6 mice. The isolated cells were differentiated into immature dendritic cells (imDCs) and then matured by lipopolysaccharide (LPS) stimulation. The matured dendritic cells (mDCs) were treated with each of the four tumor-associated antigens (TAAs: WT-1, MUC-1, BIRC5, TERT) individually and as a combined mixture of all four. Subsequently, the cytotoxicity was analyzed using Annexin V/7-AAD staining. To compare the apoptotic and necrotic cell populations, we used untreated immature or mature DCs as a negative control and H_2_O_2_-treated mature DCs as a positive control.

**Figure 1:**
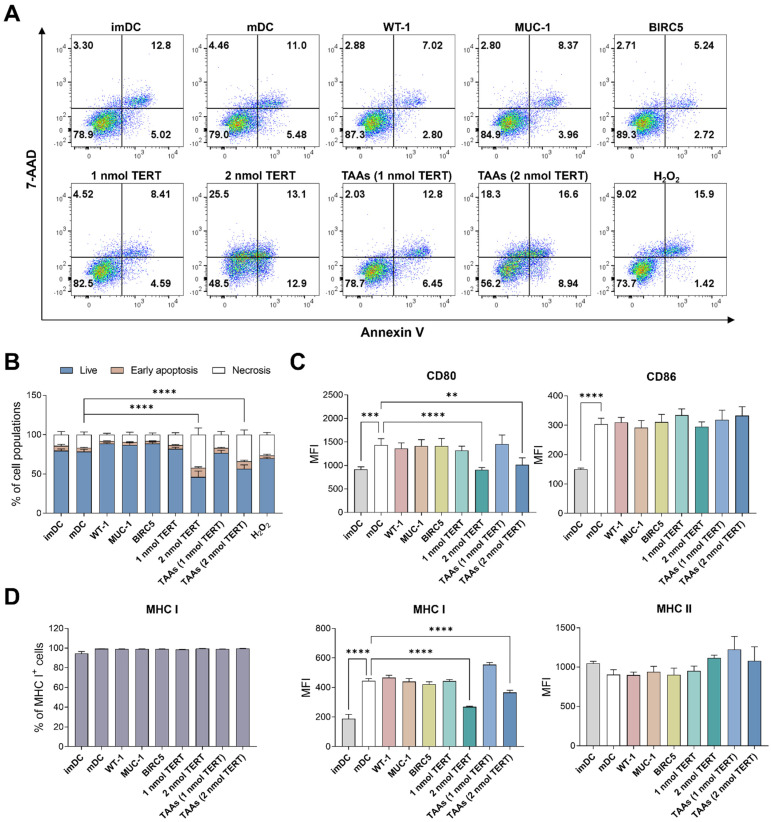
Cytotoxicity in DCs following combinational TAAs pulsing. (**A**) Cellular toxicity was analyzed using Annexin V/7-AAD staining. (**B**) The individual or combinational treatment with 2 nmol TERT increased the proportion of necrotic (7-AAD^+^) and early apoptotic (Annexin V^+^/7-AAD^−^) cells. (**C**) The individual or combinational treatment with 2 nmol TERT reduced the MFI levels in CD80 and MHC I expression. (**D**) There were no significant changes in the MHC I-positive population following treatment with TAAs. Results are shown as mean ± SD. ** *p* < 0.01, *** *p* < 0.001, **** *p* < 0.0001.

In our in vitro preliminary experiments, we ascertained that the optimal concentration for each WT-1, MUC-1, and BIRC5 was 2 nmol. The group treated with those three TAAs individually exhibited no significant differences compared to the negative controls, as expected. Furthermore, we also added the new antigen TERT with the same concentration of 2 nmol, which unexpectedly resulted in a marked increase in both necrosis and apoptosis compared to other TAAs. Thus, we conducted a limited-range testing to identify the non-cytotoxic concentration of TERT. We found the appropriate concentration of TERT (1 nmol) that does not induce cytotoxicity when TERT is treated alone or in combination with three other TAAs (WT-1, MUC-1, and BIRC5) (Figure 1A,B).

To evaluate the antigen-presentation potential of mDCs, we assessed the mean fluorescence intensity (MFI) levels of co-stimulatory molecules, CD80 and CD86, and the antigen-presenting molecules MHC complex using flow cytometry. CD80 and CD86 are expressed on the surface of dendritic cells and promote T-cell activation by binding to their CD28 receptors. MHC molecules on dendritic cells are essential for recognizing and eliminating cancer cells, as they present cancer-derived antigens to MHC I molecules, activating CD8^+^ T cells. In our results, the maturation of dendritic cells induced by LPS treatment increased the MFI levels of CD80 and CD86. The individual or combined treatment with 2 nmol of WT-1, MUC-1, BIRC5, and 1 nmol of TERT did not show significant changes compared to mDCs. However, treatment with 2 nmol TERT, either alone or in combination with other TAAs, reduced MFI levels of CD80 and MHC I (Figure 1C). Considering that the treatment with TAAs did not alter the proportion of MHC I-positive cells, the treatment with 2 nmol of TERT reduced the intensity of MHC I expression on dendritic cells (Figure 1D). Therefore, the decrease in MFI levels of MHC I is considered to be a result of cellular toxicity induced by the 2 nmol TERT treatment. The MFI levels of CD86 and MHC II remained unaffected by the TAAs in all groups.

Based on these results, we determined the optimal concentration of TAAs combination in dendritic cells that maintain their antigen-presenting ability without inducing cellular toxicity (2 nmol of WT-1, MUC-1, BIRC5, and 1 nmol of TERT). Next, we investigated whether the combinational TAAs pulsing on dendritic cells could enforce the anti-cancer effect in a Lewis Lung Cancer (LLC1) mouse model.

### 3.2. Anti-Cancer Effect of TAAs-Pulsed mDCs in LLC1 Mouse Model

**Figure 2:**
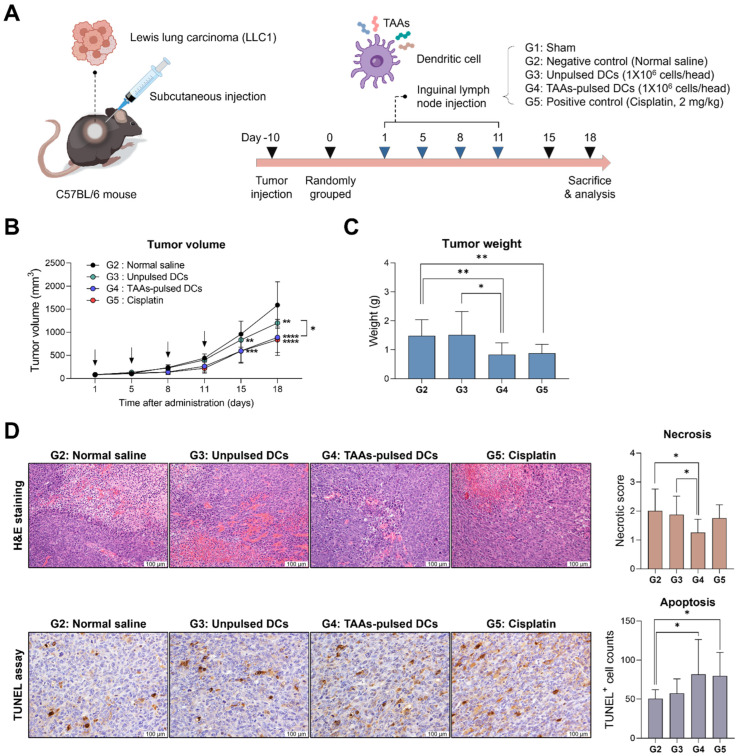
Tumor response assessment following TAAs-pulsed DCs administration. (**A**) Experimental scheme: The LLC1 cell line was transplanted to C57BL/6 mice subcutaneously, and dendritic cells were administered into the inguinal lymph nodes. (**B**,**C**) TAAs-pulsed DCs significantly reduced the tumor volumes and weights similarly to cisplatin. (**D**) H&E and TUNEL analysis showed that TAAs-pulsed DCs promote tumor-specific apoptosis. These anti-cancer effects were not observed in unpulsed DCs. Results are shown as mean ± SD. * *p* < 0.05, ** *p* < 0.01, *** *p* < 0.001, **** *p* < 0.0001.

We established an LLC1 lung carcinoma mouse model and administered mouse dendritic cells either pulsed with or without TAAs. Cisplatin, a well-known anti-cancer drug for NSCLC, was used as a positive control (Figure 2A). To evaluate the tumor growth inhibition, the change in tumor volume was calculated based on external measurements. There was a significant reduction in both TAAs-pulsed DCs (G4) and unpulsed DCs (G3) compared to the negative control treated with normal saline (G2). TAAs-pulsed DCs led to a more prominent tumor volume reduction than unpulsed DCs, showing an efficacy similar to that of the positive control (G5) (Figure 2B). The tumor tissues were isolated on day 18, and the tumor weight was measured to accurately calculate the tumor size. The TAAs-pulsed DCs showed a marked reduction in tumor weight, demonstrating efficacy comparable to the positive control (Figure 2C). Whereas the unpulsed DCs did not show a significant reduction.

To examine the histological alterations in the tumor tissues, we performed hematoxylin and eosin (H&E) staining. A tissue necrosis was scored based on the proportion of the observed necrotic areas. The TAAs-pulsed DCs (G4) significantly reduced the necrotic areas of the tumor tissues. However, the unpulsed DCs (G3) and the cisplatin did not exhibit a significant change in H&E staining. Next, we conducted a TUNEL assay to count brown-stained apoptotic cells in tumor tissue. Only both TAAs-pulsed DCs and cisplatin (G5) significantly induced tumor apoptosis (Figure 2D).

Our results showed that TAAs-pulsed DCs demonstrate remarkable tumor-suppressing ability. TAAs-pulsed DCs also showed outstanding necrosis-inhibiting potential while promoting apoptosis in tumor tissue. It suggests that TAAs-pulsed DCs may inhibit tumor growth by altering the tumor microenvironment and inducing programmed cell death via immune response.

### 3.3. Analysis of Mouse Splenic T-Cell Subpopulations

**Figure 3:**
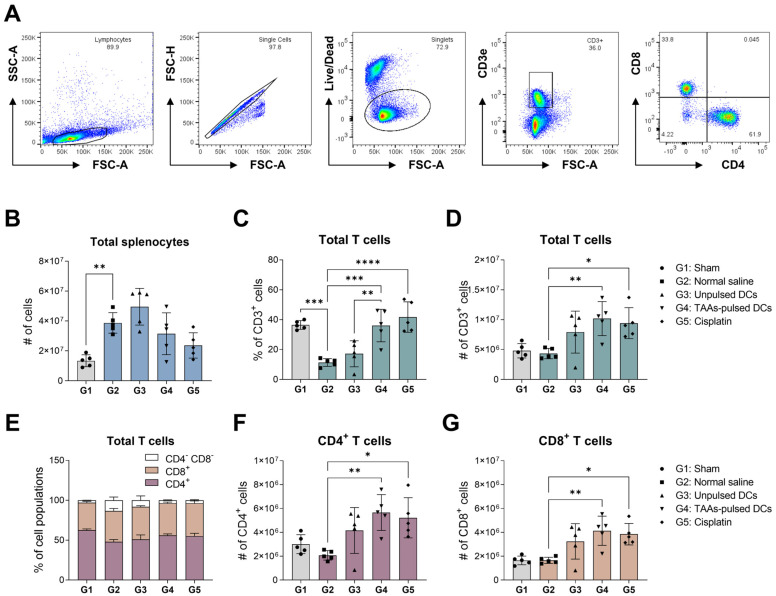
Changes in splenic T-cell subpopulations following TAAs-pulsed DCs administration. (**A**) Isolated splenocytes were first gated based on forward scatter (FSC) versus side scatter (SSC), and CD4^+^ and CD8^+^ subpopulations were identified from the CD3^+^ population. (**B**) LLC1 transplantation significantly increases the total number of splenocytes. (**C**,**D**) TAAs-pulsed DCs significantly increased the proportion and absolute number of total CD3^+^ T cells within splenocytes. (**E**) The proportion of CD4^+^ and CD8^+^ populations within CD3^+^ T cells shows no significant change. (**F**,**G**) There was a significant increase in the absolute number of CD4^+^ and CD8^+^ T cells in dendritic cells and cisplatin-administered groups, which was associated with an increase in the total number of CD3^+^ T cells. Results are shown as mean ± SD. * *p* < 0.05, ** *p* < 0.01, *** *p* < 0.001, **** *p* < 0.0001.

Dendritic cells stimulate an anti-cancer immune response by presenting antigens to T cells, activating both CD4^+^ helper and CD8^+^ cytotoxic T lymphocytes. We investigated the changes in mouse splenic T-cell populations after the dendritic cell administration. Splenocytes were isolated from the spleen of mice on day 18, and the proportions of CD3^+^, CD4^+^, and CD8^+^ T cells were measured by flow cytometry (Figure 3A). The number of total splenocytes was significantly increased after cancer cell transplantation (Figure 3B). In the negative control (G2), there was a significant decrease in the proportion of CD3^+^ T cells relative to the sham group (G1) (Figure 3C). But the absolute number of CD3^+^ T cells remained unchanged (Figure 3D). This implies that the cancer cell transplantation led to an elevation of unidentified splenocytes. Recent studies have reported that transplanted cancer cells can augment the population of immunosuppressive cells like myeloid-derived suppressor cells (MDSCs) as an evasion mechanism against the immune system [25]. The groups administered with dendritic cells showed a tendency to increase the proportion and number of CD3^+^ T cells (Figure 3C,D). Unpulsed DCs (G3) did not demonstrate statistical significance. However, TAAs-pulsed DCs and cisplatin (G4, G5) significantly increased the proportion and number of CD3^+^ T cells compared to the negative control. Our results indicate that the combinational TAAs pulsing on DCs promote the proliferation and accumulation of CD3^+^ T cells by presenting antigens to T cells more effectively.

In the analysis of the proportions of CD4^+^ and CD8^+^ subpopulations, no significant changes were observed in all experimental groups (Figure 3E). However, the DC-administered groups (G3, G4) showed a significant increase in the absolute numbers of CD4^+^ and CD8^+^ T cells. These results seem to be related to the increase in the absolute number of CD3^+^ T cells following dendritic cell administration (Figure 3F,G).

### 3.4. IFN-γ Expression in Mouse Splenic CD4^+^ and CD8^+^ T Cells

**Figure 4:**
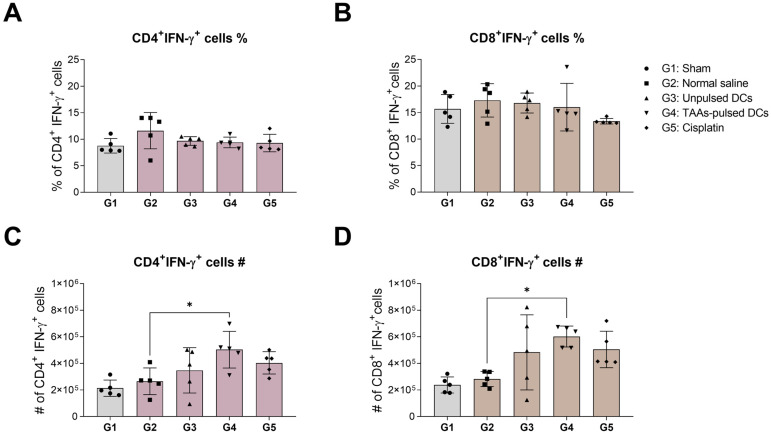
IFN-γ expression levels in splenic CD4^+^ and CD8^+^ T cells following TAAs-pulsed DCs administration. Isolated splenocytes were co-stained with IFN-γ specific fluorescent monoclonal antibody and analyzed by flow cytometry. (**A**,**B**) There were no significant differences in the proportions of CD4^+^ and CD8^+^ T cells expressing IFN-γ across all groups. (**C**,**D**) Only the TAAs-pulsed DCs significantly increased the absolute numbers of CD4^+^ and CD8^+^ T cells expressing IFN-γ compared to negative control. Results are shown as mean ± SD. * *p* < 0.05.

Interferon-gamma (IFN-γ) is a crucial cytokine secreted by Th1 cells or cytokine-producing T cells. IFN-γ plays a pivotal role in immune response and contributes to the elimination of cancer cells. We investigated the impact of TAAs-pulsed DCs on IFN-γ expressing T-cell subpopulation. The proportion and number of IFN-γ expressing CD4^+^ and CD8^+^ T cells were stained with IFN-γ intracellular marker and analyzed using flow cytometry. The results showed no changes in the proportion of IFN-γ expressing T-cell subpopulations in all groups (Figure 4A,B). However, the absolute number of both IFN-γ expressing CD4^+^ and CD8^+^ T cells significantly increased after TAAs-pulsed DCs administration (Figure 4C,D).

### 3.5. In Vitro Proliferation and IFN-γ Secretion of Mouse Splenocytes

**Figure 5:**
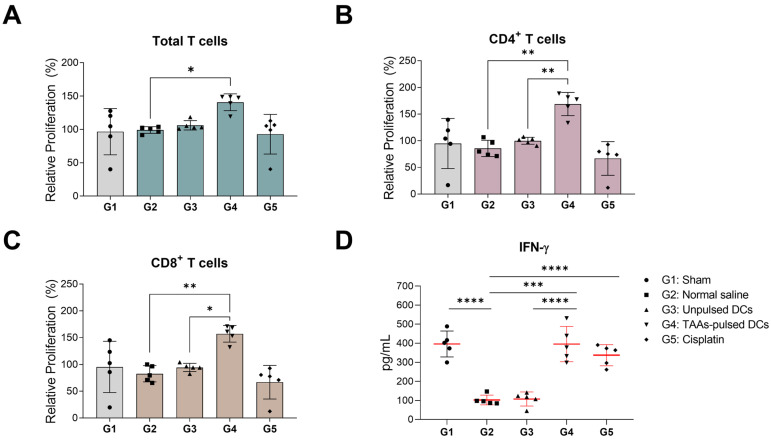
In vitro proliferation and IFN-γ secretion of isolated splenocytes after TAAs-pulsed DCs administration. Isolated splenocytes from mice were stained with CTV and further activated by PMA/Ionomycin for 3 days. Relative proliferation and IFN-γ secretion levels were analyzed using flow cytometry and ELISA. (**A**–**C**) Significant activation and proliferation were observed only in the CD3^+^, CD4^+^, and CD8^+^ splenic T cells of mice administered with TAAs-pulsed DCs. (**D**) IFN-γ secretion was significantly restored in the splenocytes of mice administered with TAAs-pulsed DCs and cisplatin. Results are shown as mean ± SD. * *p* < 0.05, ** *p* < 0.01, *** *p* < 0.001, **** *p* < 0.0001.

T cells often exhibit reduced expression and secretion of IFN-γ in cancer patients [26]. Thus, we investigated whether TAAs-pulsed DCs not only augment T-cell proliferation but also enhance the capability to secrete IFN-γ. The isolated splenocytes were stained with Cell Trace Violet (CTV) and activated with PMA and ionomycin. The proliferation rate of activated T cells was analyzed using flow cytometry. Significant in vitro proliferation of T cells was observed only in the splenocytes of the TAAs-pulsed DCs administered group (G4) (Figure 5A). The group administered with TAAs-pulsed DCs showed remarkably higher proliferation in the CD4^+^ and CD8^+^ subpopulations than those in the unpulsed DCs administered group (G3) (Figure 5B,C). However, in the cisplatin-administered group (G5), there were no significant changes compared to the sham (G1) and negative control groups (G2).

Next, IFN-γ secretion of activated splenocytes was quantified by ELISA analysis using the supernatants. As the effect of the cancer transplantation, IFN-γ secretion in the negative control (G2) was significantly decreased compared to the sham group (G1). The unpulsed DCs (G3) failed to restore the IFN-γ secretion levels, while the TAAs-pulsed DCs (G4) and cisplatin (G5) significantly restored the IFN-γ secretion levels. These results indicate that combinational pulsing of TAAs effectively enforces the DCs-based immune response and the IFN-γ secretion capability against cancer cells (Figure 5D).

## 4. Discussion

Non-small cell lung cancer (NSCLC) remains a formidable challenge in the realm of cancer, marked by high mortality rates despite significant advances in diagnostics and therapies [27]. The diversified treatment approaches for lung cancer, encompassing chemotherapy, radiotherapy, targeted therapy, and immunotherapy, highlight the complexity of combating this disease [4]. The advent of mutation-targeting drugs in the last decade presents a promising but contested avenue, given the emerging issue of drug resistance. In response, the rising importance of combination therapies, such as PD-1/PD-L1 inhibitors, underscores the necessity of activating the immune system to enhance treatment efficacy. Dendritic cells, recognized for their regulatory roles in bridging innate and adaptive immunity, have gained prominence in therapeutic applications due to their antigen-capturing and presenting capabilities. The FDA-approved dendritic cell-based therapy, Sipuleucel-T, has demonstrated a prolonged immune stimulation effect in metastatic castration-resistant prostate cancer (mCRPC) [28,29]. However, its limited extension of survival compared to conventional chemotherapy reveals existing constraints. Ongoing research explores strategies to augment dendritic cell functions, including antigen presentation, activation capabilities, and the application of diverse antigens tailored to different cancer types.

In our study, we focused on enhancing dendritic cell immunotherapy by combining four tumor-specific antigens (TAAs) expressed in NSCLC. The addition of TERT to the preliminary selection of three TAAs (WT-1, MUC-1, and BIRC5) and the determination of an optimal combination. In the LLC1 mouse model, TAAs-pulsed DCs demonstrated anti-cancer potential by inducing apoptosis in cancer cells and inhibiting tumor growth. Splenocyte analysis revealed a restoration of T-cell proportion, particularly an increase in CD4^+^ and CD8^+^ T cells expressing IFN-γ. The in vitro activation assays using splenocytes exhibited a specific increase in T-cell proliferation and IFN-γ secretion in the TAAs-pulsed DCs group. Notably, while cisplatin administration increased the total number of splenic T cells, it did not induce significant proliferation under in vitro conditions, emphasizing the complexity of in vivo and in vitro interactions. In contrast, the observed increase in in vitro T-cell proliferation within the TAAs-pulsed DCs group is believed to be linked to the pre-existing population of memory T cells in the spleen following dendritic cell administration [30]. This indicates that the administration of TAAs-pulsed DCs may have preconditioned the immune system, particularly by enhancing the memory T-cell response. These findings highlight the potential of combinational TAAs pulsing on dendritic cells to augment the anti-cancer effect.

Previous research predominantly focused on enhancing the antigen-presenting capability of dendritic cells through the pulsing of a single cancer-specific TAA. Such studies often involve allogeneic transplantation of mouse-derived dendritic cells to assess the intricate interactions of the immune system. However, obtaining a sufficient number of allogeneic mouse-derived dendritic cells for comparing single TAA or various TAA combinations presents a notable challenge. Consequently, additional screening remains necessary to identify the optimal number of TAAs, their concentrations, and combinations. In a recent study by Yoshitake et al., the efficacy of anti-cancer therapy was assessed by administering a vaccine containing a mixture of three TAA peptides (LY6K, CDCA1, and IMP-3) to patients with head and neck squamous cell cancer [31]. The study observed an increase in cytotoxic T lymphocytes (CTLs) specific to the administered TAAs in the patients’ blood, leading to a prolonged overall survival period as the number of CTLs responsive to TAAs increased. Our current study takes a comprehensive approach, examining the response of CD3^+^, CD4^+^, and CD8^+^ T cells within the spleen. As the CTL response specific to TAAs may reveal a more intricate immune mechanism, further investigation is necessary to understand how the antigen-specific CTL responses to dendritic cells depend on the type, number, and combination of TAAs.

## 5. Conclusions

This study focuses on the anti-cancer effects of pulsing dendritic cells (DCs) with a combination of four TAAs for cancer immunotherapy. Our findings reveal that TAAs-pulsed DCs exhibit significant anti-cancer effects in the LLC1 mouse model, characterized by suppressed tumor growth and an augmented splenic T-cell population. Additionally, we observed a notable improvement in the proliferative capacity of T cells and an increase in IFN-γ secretion in vitro, underscoring the potential of this combinational approach for enhancing dendritic cell therapies in cancer treatment. This study lays the groundwork for optimizing dendritic cell-based immunotherapies, emphasizing the need for continued investigation to refine and tailor these approaches for different cancer types. Future studies should focus on maximizing therapeutic effects through the development of more sophisticated TAA combinations and further advancements in dendritic cell-based therapies.

## Data Availability

The data used in this study are available from the corresponding author upon reasonable request.
